# Greater Auricular Neuropathy in Hansen’s Disease

**DOI:** 10.4269/ajtmh.19-0488

**Published:** 2019-10

**Authors:** Suryanarayana Sharma, Amit Kulkarni, Vijay K. Sharma

**Affiliations:** 1 Division of Neurology, National University Health System, Singapore;; 2 BGS Global Hospitals, Bangalore, India;; 3 Sagar Hospital, Bangalore, India;; 4 Yong Loo Lin School of Medicine, National University of Singapore, Singapore

## CASE DESCRIPTION

A 28-year-old Indian man presented with painless and progressive swelling on the right side of his neck for the past 2 years. He denied a history of neck injury, prolonged fever, loss of appetite or weight, skin rashes, or diabetes mellitus.

**Figure 1.**
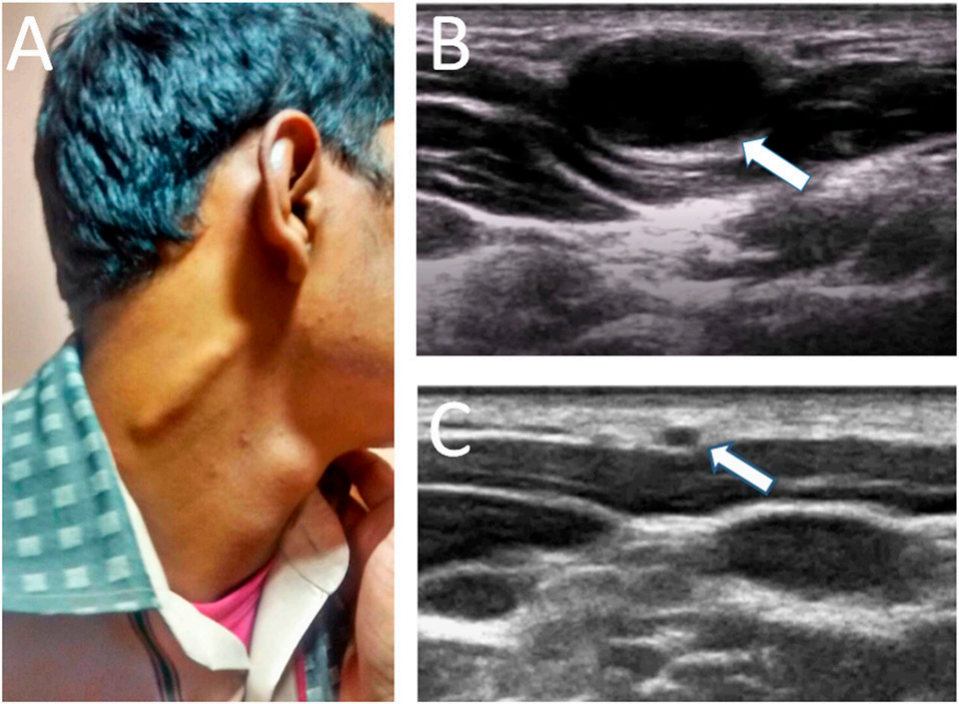
Clinical and ultrasound findings in greater auricular neuropathy (GAN) in Hansen’s disease. (**A**) Cord-like enlargement of the right GAN. Thickening of the right GAN was confirmed on B-mode high-resolution ultrasonography (**B**). Color Doppler ulrasononography revealed increased perineural vascularity. For reference, the normal ultrasonographic appearance of the left GAN is shown in panel **C**. This figure appears in color at www.ajtmh.org.

Examination revealed a cord-like, nontender swelling on the right neck (Figure 1A), which could be moved side-to-side. Systemic examination was unremarkable.

Blood counts, erythrocyte sedimentation rate, blood sugar, and chest X-ray were normal. Mantoux test was negative. A slit skin smear from the right ear lobe showed a large number of acid fast bacilli in the nerves He refused fine needle aspiration cytology or punch biopsy of the nerve.

High-resolution ultrasound revealed diffuse thickening of the right greater auricular nerve (GAN) with diffuse fascicular enlargement (Figure 1B, diameter 4.6 cm; normal 0.14 ± 0.03 cm).^1^ Imaging of the left GAN was normal (Figure 1C). Nerve ultrasound and electrophysiological studies of other peripheral nerves (including ulnar nerves) were unremarkable.

With multidrug treatment for leprosy, he remained asymptomatic at 3-month outpatient visit. He did not come for further follow-up.

## DISCUSSION

The GAN is a large sensory nerve in the posterior triangle of the neck, traversing upward and parallel to the external jugular vein. It is the commonest pure sensory cutaneous nerve affected in leprosy.^2^ Commonest differential diagnoses are thrombosed external jugular vein, enlarged cervical lymph nodes, cold abscess of the nerve in tuberculosis and other granulomatous diseases, traumatic neuromas, tumorous infiltrations, and Charcot–Marie–Tooth disease.

High-resolution nerve ultrasound is a noninvasive imaging tool for evaluating nerve thickening, edema, inflammation, abscess, fascicular enlargement, and secondary involvement in lepra reaction.^3^ Color Doppler helps in assessing endoneural vascularity as an indirect marker for inflammation. Furthermore, ultrasound can guide the nerve biopsy for a better yield and help in early diagnosis and treatment as well as monitoring the therapeutic response.
